# Effects of Cashew Nuts (*Anacardium occidentale* L.) and Cashew Nut Oil on Intestinal Permeability and Inflammatory Markers during an Energy-Restricted 8-Week Intervention: A Randomized Controlled Trial (Brazilian Nuts Study)

**DOI:** 10.3390/foods13182917

**Published:** 2024-09-14

**Authors:** Talitha Silva Meneguelli, Aline Lage Wendling, Ana Claudia Pelissari Kravchychyn, Daniela Mayumi Usuda Prado Rocha, Ana Paula Dionísio, Josefina Bressan, Hércia Stampini Duarte Martino, Elad Tako, Helen Hermana Miranda Hermsdorff

**Affiliations:** 1 Laboratory of Clinical Analysis and Genomics (LACEG), Department of Nutrition and Health, Universidade Federal de Viçosa, Viçosa 36570-200, MG, Brazil; talithasilvameneguelli@gmail.com (T.S.M.); aline.wendling@ufv.br (A.L.W.); ana.pelissari@ufv.br (A.C.P.K.); daniela.rocha@ufv.br (D.M.U.P.R.); jbrm@ufv.br (J.B.); 2 Laboratory of Energy Metabolism and Body Composition (LAMECC), Department of Nutrition and Health, Universidade Federal de Viçosa, Viçosa 36570-200, MG, Brazil; 3 Brazilian Agricultural Research Corporation (Embrapa) Agroindústria Tropical—CNPAT, Fortaleza 60511-110, CE, Brazil; ana.dionisio@embrapa.br; 4 Laboratory of Experimental Nutrition, Department of Nutrition and Health, Universidade Federal de Viçosa, Viçosa 36570-200, MG, Brazil; hercia@ufv.br; 5 Trace Minerals and Nutrition Laboratory, Department of Food Science, Cornell University, Ithaca, NY 14850, USA; et79@cornell.edu

**Keywords:** *Anacardium occidentale* L., cashew nut oil, intestinal permeability, inflammation, energy-restricted diet

## Abstract

Cashew nuts can contribute to improving intestinal permeability and inflammation as they contain essential nutrients and bioactive compounds, but no clinical trials have evaluated these potential effects. This randomized trial aimed to assess the effects of cashew nuts and their oil on intestinal permeability and inflammatory markers. Sixty-four adults with overweight or obesity were allocated into three groups receiving energy restriction (−500 kcal/day): control (CT, free nuts), cashew nuts (CN, 30 g/day), or cashew nut oil (OL, 30 mL/day). Urine lactulose and mannitol, plasma zonulin and the lipopolysaccharide-binding protein (LBP), plasma interleukins (IL-6, TNF-α, IL-10, IL-1β, IL-8, and IL-12p70), and C-reactive proteins were analyzed. Energy restriction reduced body fat and other indicators of adiposity without differences between the groups. Only the control group increased LBPs after an 8-week intervention. There were no statistically significant differences found between the groups in terms of intestinal permeability and inflammatory markers. In conclusion, incorporating cashew nuts or cashew nut oil into an energy-restricted 8-week dietary intervention did not change intestinal permeability and inflammatory markers. As studies evaluating cashew nuts on these markers remain scarce, further research is needed, perhaps with a longer study period and a higher concentration of cashew nuts and oil.

## 1. Introduction

Intestinal permeability, a functional aspect of the intestinal barrier, is influenced by the integrity of the epithelial layer and is measured by analyzing flux rates across the intestinal wall. Epithelial cells are connected by junction complexes, including tight junctions, adherents’ junctions, and desmosomes [1]. Tight junctions, characterized by proteins like occludins, claudins, and zonulin regulate the passage of molecules through the paracellular space [2]. Elevated intestinal permeability occurs when tight junctions between enterocytes fail to effectively prevent the entry of microbes or toxins into the systemic circulation [3].

Individuals with obesity have presented increased intestinal permeability, allowing toxins and bacteria to easily enter the bloodstream [4]. Multiple factors, including chronic inflammation and changes in gut microbiota composition, may contribute to altered intestinal permeability among individuals with this condition [5].

The increase in intestinal permeability can lead to “metabolic endotoxemia”, where bacterial components like lipopolysaccharides (LPS) bind to the lipopolysaccharide-binding protein (LBP), triggering an inflammatory response [6]. Interestingly, while directly measuring bacterial products like LPS in biological fluids has limitations, LBP levels are recommended as a clinical marker for evaluating “effective endotoxemia” [7]. Unlike other acute phase reactants, LBPs increase gradually, enabling monitoring of the interaction between bacterial components, particularly LPS, and innate immune cells over time [7]. Furthermore, low-grade inflammation is a recognized link between obesity and other chronic conditions like insulin resistance, type 2 diabetes, cardiovascular diseases, and metabolic disorders [6].

Weight reduction is one of the most effective treatments for obesity [8], and it also improves metabolic disturbances while reducing systemic inflammatory tone [9,10]. Cashew nuts possess properties that promote weight loss and enhance intestinal permeability [11,12]. They are particularly abundant in monounsaturated fatty acids (MUFA), with oleic acid constituting the majority at 61.8%, and they contain a diverse array of essential nutrients and bioactive compounds, including dietary fibers, minerals, carotenoids, phytosterols, amino acids, and flavonoids like catechin, epicatechin, and epigallocatechin [13,14,15]. Moreover, cashew nut oil appears to be promising for health, as it shares properties akin to extra-virgin olive oil, such as being rich in MUFA, primarily oleic acid.

However, few studies have evaluated whether an energy-restricted diet also affects intestinal permeability [16,17]. Only one study, from our research group, has evaluated the effects of nuts on intestinal permeability [11]. Our group still evaluated the effects of these foods on body composition, endothelial function, and satiety [12,18,19,20]. Apart from our study, there is currently no study assessing the impact of cashew nuts on intestinal permeability, nor is there a clinical trial evaluating their effects on inflammation.

Thus, this study aims to evaluate the effects of consuming cashew nuts (*Anacardium occidentale* L.) and its oil, associated with an 8-week energy-restricted diet on intestinal permeability and inflammatory markers. Our hypothesis suggests that both cashew nuts and cashew nut oil associated with an energy-restricted diet have the potential to enhance intestinal permeability and improve inflammation in these individuals.

## 2. Materials and Methods

### 2.1. Study Design

This study is an 8-week randomized controlled intervention, performed at the Department of Nutrition and Health of the Universidade Federal de Viçosa (UFV), Brazil, between January 2022 and July 2022. Participants received nutritional intervention with energy restriction (−500 kcal/day) and were allocated to one of three groups: control (CT; free nuts), cashew nuts (CN; 30 g/day), and cashew nut oil (OL; 30 mL/day). During the intervention period, participants attended the Laboratory of Energy Metabolism and Body Composition (LAMECC/UFV) on three occasions: the initial and final days for blood collection, anthropometry, body composition, and an intestinal permeability test, and in the fourth week (30 days) for a face-to-face monitoring visit and anthropometric measurements. Participants were monitored online between their in-person visits.

### 2.2. Ethical Aspects

This research adhered to the Declaration of Helsinki, and the protocols involving human participants received approval from the Ethics Committee/UFV (Approval No. 4.543.541/CEPH). Written informed consent was obtained from all individuals involved. This study is registered with the Brazilian Registry of Clinical Trials (ReBEC) under the ID RBR-8xzkyp2.

### 2.3. Study Participants

Inclusion criteria encompassed both men and women aged 20–55, with overweight (BMI 27–29.9 kg/m^2^), specific waist circumference (WC) measurements (≥80 cm for women; ≥90 cm for men), and body fat percentages (>30% for women and >20% for men). These criteria were associated with at least one other component of metabolic syndrome (MS), such as elevated triglycerides (TG ≥ 150 mg/dL), blood pressure (≥130/85 mm/Hg), fasting blood glucose (≥100 mg/dL), or the use of medication to manage these markers. Additionally, individuals with obesity (BMI ≥30 kg/m^2^), an elevated WC, and body fat (%) with or without metabolic complications, were also included.

Exclusion criteria comprised pregnant, lactating, or menopausal women; athletes; vegans; those with insulin-dependent diabetes; HIV diagnosis; digestive, hepatic, renal, cardiovascular, thyroid, cancer, inflammatory diseases, and eating disorders; a history of drug and/or alcohol abuse; aversion or allergy to nuts; recent infection; habitual consumption of nuts exceeding 30 g/day; the use of drugs like anti-inflammatories, corticosteroids, and antibiotics; five percent of weight instability in the last 3 months; alcohol consumption (≈168 g/week); and the consumption of essential nutrient supplements.

### 2.4. Randomization

Following the initial run-in period, participants were randomly assigned considering gender, age, and BMI. This method aimed to achieve an even distribution of potential influencing factors on performance outcome variables. The randomization process utilized the MinimPy 0.3 program [21].

### 2.5. Sample Size and Study Power

The sample size of this study was previously described [20]. Regarding study power, an effect size of 0.27 was derived from an eta-squared value of 0.068 using intestinal permeability data from our database. This analysis included three groups, two intervention points, an alpha level of 0.05, and a sample of 64 individuals. This resulted in a study power of 0.90 (Supplementary Figure S1).

### 2.6. Dietary Intervention

All the dietary interventions were previously described by our group [20]. The intervention groups, those receiving cashew nuts and cashew nut oil, had an additional 30 g/day and 30 mL/day included in their meal plans, respectively. Cashew nut oil was produced in Brazil by the Brazilian Agricultural Research Corporation (Embrapa) Agroindústria Tropical in Fortaleza, CE. The nuts were pre-portioned into vacuum-sealed laminated packages, while the oil was divided into amber glass bottles with a volume of 250 mL. Both products were stored at −20 °C in a freezer to preserve nutrients, prevent oxidation, maintain sensory qualities, and avoid microbiological contamination until distribution to study participants.

### 2.7. Outcomes

Body fat loss was the primary outcome, as previously published [20]. For this study, we reported the changes in intestinal permeability and inflammatory markers.

### 2.8. Anthropometry and Body Composition

Anthropometric evaluation was conducted on the initial, midway, and final days of the intervention, while body composition was obtained at the initial and final days of the intervention. The weight was determined by bioelectric impedance (Inbody 230, Biospace Corp., Cheonan-Si, Chungcheongnam-Do, Republic of Korea) with a capacity of 250 kg and an accuracy level of 100 g. The height was measured using a vertical millimeter stadiometer extending 2.2 m, with a precision of 0.5 cm. Hip (HC), neck (NC), and waist circumferences (WC) were measured using an inelastic tape with a precision of 0.1 cm. The WC was measured at umbilical height. Dual-energy X-ray absorptiometry (Lunar Prodigy Advance DXA System, GE Lunar, Milwaukee, WI, USA) was employed for the body composition assessment. The android region spans between the ribs and the pelvis, while the gynoid region encompasses the hips and upper thighs, overlapping with both the leg and truncal regions.

### 2.9. Food Assessment

To monitor food intake throughout the intervention, a 24-h recall (24HR) was administered at the beginning and end of this study. The 24HR-ERICA software was employed to quantify the reported intake while nutrient analysis utilized the IBGE table [22,23].

### 2.10. Intestinal Permeability Markers

The methodology for collecting and separating blood was previously described [20]. We assessed LBPs and zonulin in EDTA plasma, using human Enzyme-Linked Immunosorbent Assay kits (LBP, catalog n° MBS704355; Human zonulin, catalog n° MBS167049). All measurements were conducted with a Thermo Multiskan™ FC Microplate Photometer (Thermo Fisher Scientific, Vantaa, Finland), following the manufacturer’s instructions.

Also, the participants collected urine samples before the test, comprising all urine excreted from the last meal of the day until the test. This urine served as a control. On the following day, after an overnight fast, participants consumed a solution containing lactulose (10 g), mannitol (5 g), and sucrose (20 g) in a volume of 200 mL. After drinking this solution, urine was collected over a period of four hours and thirty minutes. Participants refrained from food intake during the test, and water consumption was regulated (150 mL after 2 h and 3 h of testing). The total volume of urine excreted the night before the test (control urine) and during the test (test urine) was documented, and thimerosal was added (4:1, mg:mL) to the samples, which were stored at −20 °C until analysis. For analyses, the urine samples were thawed and homogenized in a vortex, and 2 mL of urine was withdrawn using a sterile syringe. The syringe was connected to a polyethersulfonic (PES) microporous membrane (0.22 μm × 13 mm), allowing the sample to be filtered directly into vials for HPLC (Shimadzu, model CBM 2A, Japan). The final volume of filtered urine in the vials was 600 µL.

Lactulose and mannitol excretion in urine was measured using HPLC on a Dionex Ultimate 3000 Dual coupled to a Refractive Index (IR) Detector (Shodex RI-101, Sunnyvale, CA, USA) maintained at 40 °C. Analytes were separated on an ion exclusion column (Phenomenex Rezex ROA, 300 × 7.8 mm) at 40 °C. The mobile phase consisted of 5 mm of sulfuric acid (H_2_SO_4_) with a flow rate of 0.7 mL/min. Internal lactulose and mannitol standards were employed to establish the normalization curve. The total volume of urine collected was multiplied by the concentration of each sugar to determine the overall amount excreted in the urine. The results in the spreadsheet are expressed as the percentage excretion of mannitol (% M) and lactulose (% L) and then calculated as the lactulose/mannitol (L/M) ratio.

### 2.11. Cardiometabolic Markers

We evaluated cardiometabolic risk as glucose, insulin, triglycerides, total cholesterol, LDL-c, HDL-c, VLDL-c, as well as apolipoprotein-A-1 (APO-A-1), apolipoprotein-B (APO-B), and hepatic markers such as AST transaminase, gamma GT, ALT transaminase, and alkaline phosphatase.

### 2.12. Inflammatory Markers

The ultrasensitive method to assess plasma C-reactive protein (CRP) was performed by the Hemolab clinical analysis laboratory using immunoturbidimetry and following established protocols. Interleukins (IL-6, TNF-α, IL-10, IL-1β, IL-8, and IL-12p70) were analyzed by flow cytometry in plasma samples, using commercial kits (BD Biosciences^®^, San Jose, CA, USA) and the data analyzed in FCAP Array Software v3.0 (DB Immunocytometry Systems, San Jose, CA, USA) at the Nucleus of Microscopy and Microanalysis of the UFV.

### 2.13. Statistical Analysis

Statistical analysis was performed using SPSS version 22.0 (SPSS, Inc., Chicago, IL, USA), considering *p*-value < 0.05 as statistically significant. The Shapiro–Wilk test was performed to check the normality of variables. Data were presented as mean values and standard deviation. Comparisons between the groups were conducted using either one-way ANOVA with Tukey’s post hoc test or the non-parametric Kruskal–Wallis test with Dunn’s post hoc test. Within-group differences between baseline and post-intervention were evaluated through pairwise tests, utilizing either the paired *t*-test or Wilcoxon test. One-way repeated measures ANOVA with group and time interactions followed by post hoc testing was used to evaluate the effect of time (baseline, return, and endpoint) on the following variables: weight, waist, hip, and neck circumferences in the three groups. For qualitative variables, the chi-square test was used.

## 3. Results

Based on the baseline characteristics of the individuals, the average age was 33.42 year ± 8.78. At the end of the study, 64 individuals completed the intervention (25 men and 39 women). There were no statistically significant differences in any variables between the groups at baseline, which demonstrates homogeneity between the groups before intervention (Table 1). All participants exhibited a reduction in energy intake (−205 kcal; *p* = 0.026 for paired tests). Based on the R-24h, food consumption was previously described [20].

**Figure 1:**
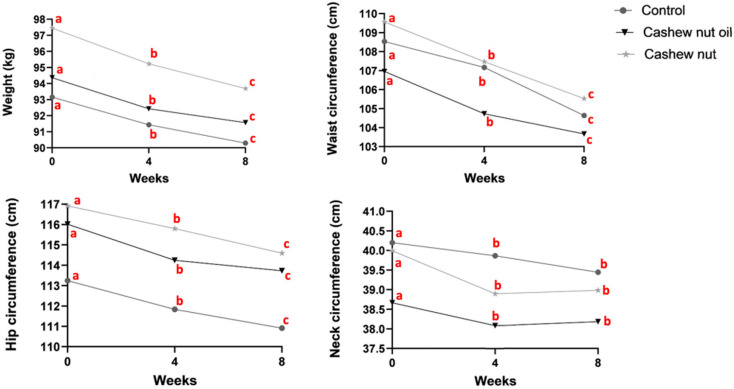
Anthropometric differences between the baseline, return, and endpoint. Different letters indicate statistical significance resulting from the one-way repeated measures ANOVA with group and time interaction.

Figure 1 illustrates the impact of three different time points: time 1 (baseline), time 2 (return), and time 3 (endpoint) on anthropometric variables such as weight, WC, HC, and neck circumference. Concerning weight, WC, and HC variables, a notable effect of the three time points (time 1, 2, and 3) was observed across all groups (control, nuts, and oil) (*p* < 0.001). However, for the neck circumference there was a significant difference between time 1 and time 2 (*p* < 0.001) and between time 1 and time 3 (*p* < 0.001), but not between time 2 and time 3 (*p* = 0.483).

When comparing outcomes related to intestinal permeability and inflammatory markers before and after the intervention, participants in the control group increased LBPs after the 8-week intervention (*p* = 0.045). No other changes were statistically significant for other intestinal permeability and inflammation markers in any of the three groups nor between groups (Table 2).

**Figure 2:**
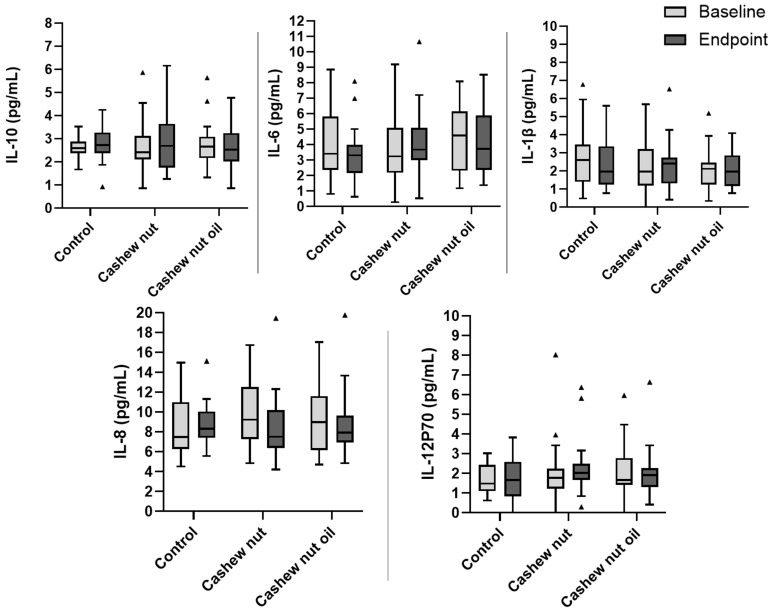
Boxplot of plasma cytokines according to groups at baseline and after 8-week energy-restricted intervention according to treatment groups. IL: interleukin. One-way ANOVA comparing the three groups at baseline and endpoint (*p* < 0.05). There were no differences between groups. Outliers are represented with the triangle symbol.

There were no changes in inflammatory markers at baseline nor endpoint in plasma concentrations of IL-12p70, IL-10, IL-6, IL-1β, and IL-8 (Figure 2). When comparing before and after treatment within each group (control, cashew nuts, and cashew nut oil), no statistically significant differences were observed in any of the three groups.

**Figure 3:**
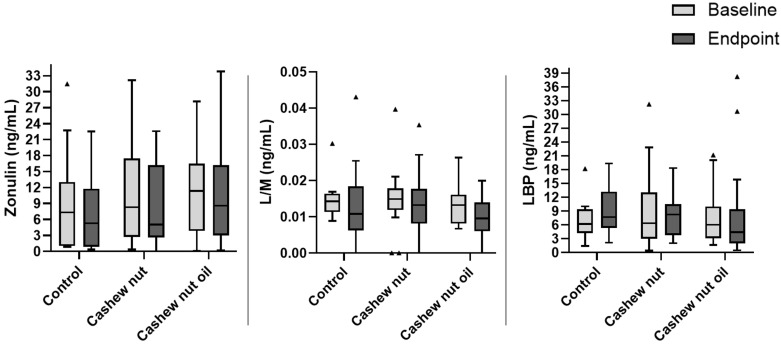
Boxplot of intestinal permeability between groups at baseline and endpoint (after 8 weeks of intervention) according to treatment groups. LBP: lipopolysaccharide-binding protein. L/M: lactulose/mannitol ratio. One-way ANOVA comparing the three groups at baseline and endpoint (*p* < 0.05). There were no differences between groups. Outliers are represented with the triangle symbol.

Related to intestinal permeability markers, we did not see changes at baseline or endpoint in plasma LBPs and zonulin, or urine concentration of L/M (Figure 3). Additionally, no differences were observed before or after treatment in any of the three groups.

## 4. Discussion

In this trial, we investigated whether the consumption of cashew nuts (30 g/day) or cashew nut oil (30 mL/day) could provide additional benefits to adults with overweight/obesity, particularly in terms of intestinal permeability and inflammatory markers. These results were compared to an energy-restricted intervention alone, which was also conducted on adults with overweight/obesity. Additionally, we investigated the interrelationships between these outcomes.

In this context, LPBs increased significantly in the control group after an energy-restricted 8-week intervention, while no significant differences were observed in the groups consuming cashew nuts or cashew nut oil. Additionally, other intestinal permeability markers (zonulin and an L/M ratio), as well as inflammatory markers, showed no significant differences before or after intervention in all three groups. This demonstrates that energy restriction and weight loss per se were also unable to improve these markers, at least over 8 weeks. Since intestinal permeability and inflammatory markers are more difficult to alter due to their molecular nature (i.e., involving complex cellular and biochemical processes), longer interventions may be necessary. Moreover, there were no statistical differences in intestinal permeability and inflammatory markers between the groups (CT, CN, and OL) at baseline and after 8 weeks of intervention (Figure 2 and Figure 3).

The LBP is regarded as a reliable indicator of intestinal barrier dysfunction and bacterial translocation because it binds to LPS and is more stable compared to other markers, making it easier to detect [24]. The presence of LBPs in the bloodstream suggests that bacterial translocation has occurred due to a compromised intestinal barrier [24], which was the case of the control group. Moreover, the detection of LBPs in the blood may indicate not only intestinal barrier dysfunction but also the increase in inflammation, as LBP facilitates the binding of LPS with CD14 e MD-2, triggering an inflammatory response and consequently the potential for the development of chronic inflammatory conditions [25,26]. Hence, LBP holds significant importance as a marker in the assessment of intestinal permeability, as its presence in the blood can offer insights into intestinal health and the risk of developing diseases linked to intestinal barrier dysfunction and systemic inflammation.

Only one clinical trial addressing the effect of nuts on intestinal permeability (by lactulose and an L/M ratio) was identified in the literature, conducted by our research group as previously described [11]. However, no study specifically evaluating LBPs or zonulin was found in the literature. We have also examined the impact of cashew nut soluble extracts on gut health using an animal model, *Gallus gallus* [27]. This study revealed no alterations in inflammatory markers (NF-κB and IL-1β), occludin (a tight junction protein), or mucin 2 (a gene responsible for mucus production) [27]. Nevertheless, improvements in intestinal morphological parameters and functionality were observed due to the upregulation of aminopeptidase (AP) gene expression [27].

Studies have investigated the impact of walnuts and walnut oil on intestinal permeability related to inflammation. One study with male C57BL/6 mice used 7% of walnut oil by weight and showed that walnut oil improved the damage score in inflamed tissue and restored colonic wall permeability in mice exposed to dextran sulfate sodium (DSS) [28]. Another study using the same animal model showed that walnut supplementation protected the colonic mucosa after injury [29]. Also, researchers analyzed fecal and colonic samples, finding that the metabolic changes induced by walnut consumption may protect against DSS-induced inflammatory tissue injury [29]. In this study, the authors highlighted that the amount provided to the animals corresponds to a daily human intake of 56.6 g (2 ounces) of walnuts, which is approximately 18% of a total daily caloric intake based on a 2000-calorie diet. This amount is much larger (almost double) than what was provided in our study.

Cashew nut oil has some major and minor components that have anti-inflammatory effects, such as oleic acids, vitamin E, tocopherol, and tocotrienol, as previously described in our paper [20]. One study showed that while SFA induced activation of the NLRP3 inflammasome and subsequent IL-1β release in macrophages, the oleic acid inhibited these [30]. Furthermore, oleoylethanolamide is a bioactive lipid produced postprandially from dietary oleic acid in the small intestine and has anti-inflammatory properties [31], including acting in the reduction in IL-1β [32]. Vitamin E, abundant in cashew nut oil, also appears to have anti-inflammatory actions, including reducing IL-1β [33]. Although the precise mechanisms through which dietary fatty acids inhibit cytokine production remain unclear, they may involve the suppression of the inflammatory process at the cyclooxygenase and lipoxygenase levels [34].

Given the scarcity of studies examining nut oil, making comparisons with findings in the literature is challenging, but data from the literature show that the effects of nuts on inflammatory markers are still contradictory.

When evaluating the impact of nuts on inflammatory markers in clinical trials, most studies lasting 8 weeks or less did not show any changes in these markers, regardless of nut type or dosage (39–60 g/day of mixed nuts for 8 weeks; 1 unit/day of Brazil nuts for 8 wk; 42 g/day of cashew nuts for 4 wk; 56 g/day of almonds for 8 wk; 30 g/day of mixed nuts for 6 weeks) [35,36,37,38,39]. Only one 8-week study reduced inflammation, but it used higher doses (39–60 g/day of mixed nuts) compared to those in our study [40]. Studies also demonstrated reductions in some inflammatory markers for longer durations, such as 12 weeks [41,42,43,44,45] or 12 months [46]. However, some 12-week studies also failed to alter inflammatory markers, despite varying nut types and dosages (30 g/day or 60 g/day of hazelnuts; 59–128 g of pistachios; 30 g/day of pecan nuts or 30 mL/d of extra-virgin olive oil; 30 g/day of mixed nuts; 45–60 g/day of peanuts or almonds) [47,48,49,50,51]. Similarly, a 16-week study using 42.5 g/day of mixed nuts did not result in inflammatory changes [52]. Notably, two exceptions were observed in studies evaluating Brazil nuts, where a 6-week trial revealed reductions in inflammatory markers among individuals aged 52–75 years at risk for colorectal cancer [53], and another indicated reductions after 30 days of single intake (20 or 50 g) among healthy volunteers [54].

Although the studies mentioned above varied in target populations and types of nuts, it is plausible that a longer study duration or higher doses of cashew nuts could produce different results compared to those observed in our study. Therefore, extending the study period may be interesting to explore whether the cashew nuts can affect inflammatory markers and alter intestinal permeability.

Our study has some limitations. Firstly, we used only one 24-h recall to assess food consumption. Furthermore, study participants, all with obesity conditions, typically underestimate their food intake, further complicating the accuracy of the 24-h recall method. Moreover, the relatively short duration of this study may have constrained our findings. A longer study duration could potentially reveal the effects of cashew nuts and cashew nut oil on inflammatory and intestinal permeability markers that were not detected in eight weeks.

As for strengths, we emphasize our monitoring of participants throughout the study. In addition to conducting in-person and online visits, we maintained an open channel for participant inquiries and regularly provided booklets and information on healthy lifestyle habits to enhance adherence. In particular, this study marks the first evaluation of the impact of a nut on LBPs and zonulin, with no prior research exploring the effects of nuts on these markers of intestinal permeability. Additionally, our research group is the first to investigate the health effects of consuming cashew nut oil. Finally, the inclusion of both men and women allows for the extrapolation of our study findings to an adult population grappling with overweight and obesity. Thus, our study paves the way for future research on the impact of nuts on intestinal permeability markers that have not yet been explored, such as zonulin and LBPs. Additionally, it calls for investigations into the effects of cashew nuts on intestinal permeability and cashew nut oil on overall health.

## 5. Conclusions

Our hypothesis that cashew nuts and cashew nut oil would improve inflammation and intestinal permeability was not supported, as there was no statistically significant difference between the three groups (*p*-interindividual). We believe that some factors, such as a longer study period, higher doses of cashew nuts and cashew nut oil, and greater energy restriction, could show the benefits of cashew nuts and cashew nut oil compared to the control in improving intestinal permeability and inflammation. As this was the first study to evaluate the consumption of cashew nut and cashew nut oil on intestinal permeability and inflammation, and since cashew nut oil is a novel product not yet available on the market, further research exploring these factors individually or in combination would be valuable to determine if the results might differ.

## Figures and Tables

**Table 1:** Baseline characteristics of samples according to energy-restricted intervention groups.

Variables	Total	Control (n = 17)	Cashew Nuts (n = 24)	Cashew Nut Oil (n = 23)	*p*-Value
Age (years)	33.4 ± 8.8	34.2 ± 10.0	33.8 ± 8.4	32.5 ± 8.5	0.810
Sex					
Man (n/%)	25 (39.06)	8 (12.5)	9 (14.1)	8 (12.5)	0.720
Woman (n/%)	39 (60.94)	9 (14.1)	15 (23.4)	15 (23.4)	
Smoking					
Yes (n/%)	3 (4.7)	1 (1.6)	1 (1.6)	1 (1.6)	0.960
No (n/%)	61 (95.3)	16 (25)	23 (35.9)	22 (34.4)	
Physically active					
Yes (n/%)	22 (34.4)	4 (6.3)	10 (15.6)	8 (12.5)	0.480
No (n/%)	42 (65.6)	13 (20.3)	14 (21.9)	15 (23.4)	
Adiposity markers					
Weight (kg)	95.1 ± 1.9	92.6 ± 14.9	96.1 ± 14.8	95.3 ± 13.9	0.742
HC (cm)	116.2 ± 0.8	113.5 ± 5.9	116.6 ± 7.3	116.9 ± 6.2	0.226
WC (cm)	108.6 ± 1.4	108.1 ± 8.5	109.3 ± 11.7	107.6 ± 12.1	0.875
NC (cm)	39.6 ± 0.6	40.2 ± 3.5	39.9 ± 4.5	38.9 ± 4.6	0.613
Body fat (%)	43.4 ± 0.9	40.9 ± 8.2	43.6 ± 7.6	43.7 ± 7.9	0.471
Android (%)	50.7 ± 0.8	47.5 ± 7.5	48.1 ± 7.6	48.2 ± 8.5	0.965
Gynoid (%)	48.4 ± 1.0	42.9 ± 9.4	46.4 ± 9.1	48.7 ± 9.5	0.173
Android/Gynoid	1.1 ± 0.0	1.1 ± 0.2	1.1 ± 0.1	1.0 ± 0.1	0.055
Cardiometabolic markers					
Glucose (mg/dL)	94.6 ± 1.7	97.1 ± 18.1	91.8 ± 9.2	94.8 ± 12.5	0.449
Insulin (µUI/mL)	15.4 ± 1.0	16.8 ± 6.9	17.1 ± 10.2	12.3 ± 4.9	0.073
Triglycerides (mg/dL)	146.8 ± 11.1	175.7 ± 100.2	127.9 ± 58.9	145.8 ± 91.3	0.203
Cholesterol (mg/dL)	196.4 ± 5.1	195.1 ± 31.2	186.2 ± 31.6	207.3 ± 47.8	0.172
LDL-c (mg/dL)	111.3 ± 4.3	104.9 ± 29.7	105.9 ± 30.1	120.1 ± 35.5	0.216
HDL-c (mg/dL)	55.6 ± 1.3	55.5 ± 10.9	54.3 ± 9.8	57.9 ± 11.7	0.506
VLDL-c (mg/dL)	29.5 ± 2.2	35.1 ± 20.0	25.8 ± 11.4	29.2 ± 18.3	0.215
Apo A (mg/dL)	124.4 ± 2.1	126.9 ± 18.5	120.8 ± 12.1	128.1 ± 20.0	0.299
Apo B (mg/dL)	88.9 ± 2.7	88.7 ± 15.5	84.7 ± 17.4	93.0 ± 25.2	0.373
Intestinal permeability markers					
LBP	7.6 ± 0.8	6.9 ± 3.8	8.7 ± 7.6	7.4 ± 5.7	0.629
L/M	0.02 ± 0.01	0.04 ± 0.08	0.02 ± 0.02	0.02 ± 0.02	0.215
Inflammatory markers					
CRP (mg/L)	3.0 ± 0.4	2.3 ± 1.8	3.1 ± 2.9	3.5 ± 3.5	0.396
IL-12p70 (pg/mL)	1.9 ± 0.2	1.7 ± 0.8	1.9 ± 1.6	2.0 ± 1.3	0.746
TNF-a (pg/mL)	0.4 ± 0.2	0.3 ± 1.1	0.7 ± 1.9	0.5 ± 1.7	0.712
IL-10 (pg/mL)	2.9 ± 0.3	2.6 ± 0.5	3.3 ± 3.8	2.8 ± 0.9	0.578
IL-6 (pg/mL)	4.1 ± 0.3	3.9 ± 2.2	4.2 ± 3.2	4.2 ± 2.2	0.938
IL-1b (pg/mL)	2.3 ± 0.2	2.7 ± 1.8	2.3 ± 1.5	2.2 ± 1.1	0.521
IL-8 (pg/mL)	10.8 ± 1.3	8.5 ± 3.1	13.6 ± 15.6	9.3 ± 3.6	0.203

For quantitative variables, a one-way ANOVA followed by Tukey’s post hoc test were used, and the results are represented by mean ± SD (standard deviation). For qualitative variables, the chi-square test was used, and the results are presented in absolute and relative frequency values in Table 1 as n (%): LBP—Lipopolysaccharide-binding protein; HC—Hip circumference; WC—Waist circumference; NC—Neck circumference; LDL-c—Low-density lipoprotein-cholesterol; HDL-c—High-density lipoprotein-cholesterol; VLDL-c: Very low-density lipoprotein-cholesterol; AST—Aspartate transferase; ALT—Alanine transaminase; GGT—Gamma-glutamyl transferase; Apo—Apolipoprotein; CRP—C-reactive protein; TNF—Tumor necrosis factor; and IL—Interleukin. We used chi-square for the qualitative variable.

**Table 2:** Change in intestinal permeability and inflammatory markers according to 8-week energy-restricted intervention groups.

Outcomes		Baseline	Endpoint	Δ	*p*-Value
LBP (ng/mL)	CT	6.9 ± 3.8	9.3 ± 5.3	2.3 ± 4.4	0.045
	CN	8.7 ± 7.6	8.4 ± 4.8	−0.3 ± 6.4	0.813
	OL	7.4 ± 5.7	7.8 ± 9.4	0.4 ± 8.0	0.823
*p*-value		0.629	0.795	0.447	
Zonulin (ng/mL)	CT	8.8 ± 8.5	6.5 ± 6.8	−2.2 ± 6.2	0.160
	CN	10.6 ± 8.9	8.9 ± 7.9	−1.7 ± 8.2	0.332
	OL	11.8 ± 8.3	10.8 ± 9.5	−1.0 ± 5.6	0.389
*p*-value		0.539	0.276	0.857	
L/M	CT	0.04 ± 0.07	0.01 ± 0.01	−0.03 ± 0.08	0.170
	CN	0.02 ± 0.02	0.03 ± 0.06	0.01 ± 0.06	0.328
	OL	0.02 ± 0.02	0.02 ± 0.02	−0.003 ± 0.03	0.630
*p*-value		0.277	0.247	0.105	
IL-12P70 (pg/mL)	CT	1.7 ± 0.7	1.7 ± 1.1	0.02 ± 1.31	0.948
	CN	1.9 ± 1.6	2.3 ± 1.3	0.3 ± 1.2	0.206
	OL	2.1 ± 1.3	2.2 ± 1.4	0.1 ± 1.1	0.715
*p*-value		0.543	0.266	0.688	
TNF-α (pg/mL)	CT	0.2 ± 1.0	0.1 ± 0.2	−0.2 ± 1.0	0.457
	CN	0.7 ± 1.9	1.1 ± 3.1	0.4 ± 1.5	0.233
	OL	0.5 ± 1.6	0.5 ± 1.1	−0.02 ± 1.4	0.949
*p*-value		0.647	0.228	0.391	
IL-10 (pg/mL)	CT	2.9 ± 1.1	3.6 ± 3.9	0.7 ± 3.1	0.362
	CN	3.3 ± 3.8	2.8 ± 1.2	−0.5 ± 3.3	0.454
	OL	2.8 ± 0.9	2.7 ± 0.9	−0.1 ± 1.1	0.401
*p*-value		0.694	0.409	0.343	
IL-6 (pg/mL)	CT	3.7 ± 2.1	3.3 ± 1.9	−0.4 ± 2.4	0.504
	CN	4.2 ± 3.2	4.4 ± 2.9	0.3 ± 3.2	0.687
	OL	4.2 ± 2.1	4.1 ± 1.9	−0.1 ± 2.8	0.847
*p*-value		0.803	0.292	0.756	
IL-1β (pg/mL)	CT	2.8 ± 1.7	2.3 ± 1.4	−0.5 ± 1.0	0.056
	CN	2.3 ± 1.5	2.3 ± 1.4	0.03 ± 1.2	0.899
	OL	2.2 ± 1.1	2.03 ± 0.9	−0.2 ± 0.9	0.401
*p*-value		0.422	0.680	0.307	
IL-8 (pg/mL)	CT	8.7 ± 3.2	8.7 ± 2.3	−0.02 ± 3.6	0.980
	CN	13.6 ± 15.6	8.6 ± 3.2	−5.05 ± 15.6	0.127
	OL	9.3 ± 3.4	8.6 ± 3.05	−0.7 ± 4.1	0.376
*p*-value		0.186	0.988	0.189	

L/M: lactulose/mannitol ratio; LBP: Lipopolysaccharide-binding protein; CT: Control group; CN: Cashew nuts group; and OL: Cashew nut oil group. *p*-values were from paired *t*-tests to compare before and after intervention and a one-way ANOVA was used to compare between groups.

## Data Availability

The original contributions presented in the study are included in the article/Supplementary Materials, further inquiries can be directed to the corresponding author.

## Supplementary Materials

The following supporting information can be downloaded at: https://www.mdpi.com/article/10.3390/foods13182917/s1, Figure S1: Study power calculation according to G*Power 3.1 program.
