# Supplementary figures and images for: Effects of Cashew Nuts (Anacardium occidentale L.) and Cashew Nut Oil on Intestinal Permeability and Inflammatory Markers during an Energy-Restricted 8-Week Intervention: A Randomized Controlled Trial (Brazilian Nuts Study)
Source: Foods. 2024 Sep 14;13(18):2917. doi: 10.3390/foods13182917 (PMC11431763; doi:10.3390/foods13182917)

**Supplementary Figure S1.** Study power calculation according to G\*Power 3.1 program.

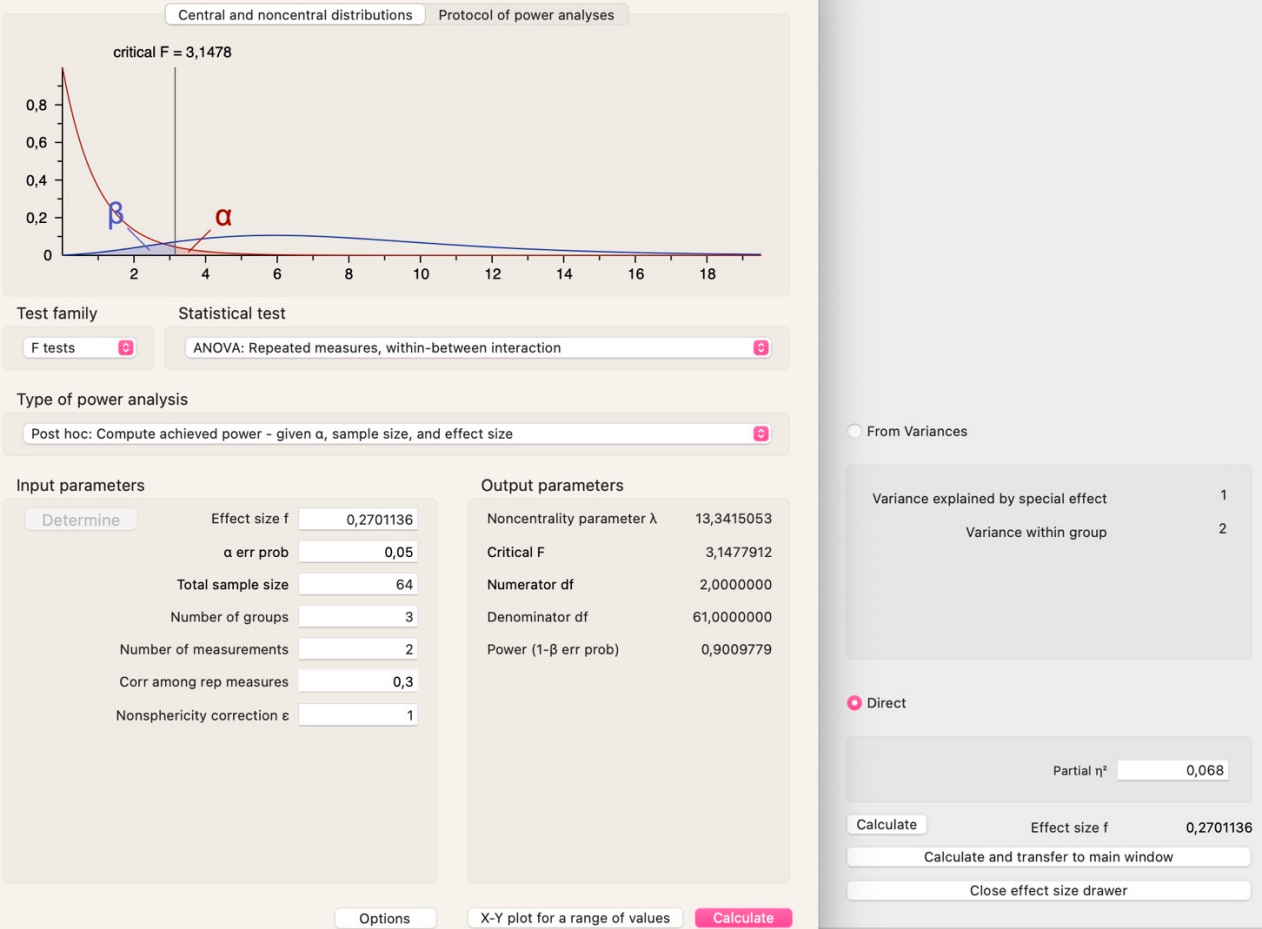

Supplement: Supplementary file 1 [file foods-13-02917-s001.zip › foods-3178291-supplementary.pdf]
